# Lightweight Design of Aircraft Double-Lug Joint Structure Based on Topology Optimization and Honeycomb Materials

**DOI:** 10.3390/ma18235339

**Published:** 2025-11-27

**Authors:** Haifeng Ou, Shumeng Pang, Weijun Tao, Yiquan Huang

**Affiliations:** 1 School of Environment and Civil Engineering, Dongguan University of Technology, Dongguan 523808, China; 2 State Key Laboratory of Explosion Science and Safety Protection, Beijing Institute of Technology, Beijing 100081, China; 3 Earthquake Engineering Research & Test Center, Guangzhou University, Guangzhou 510006, China

**Keywords:** double-lug joint structure, lightweight design, topological optimization, honeycomb material

## Abstract

In aerospace engineering, structural lightweight remains one of the core design objectives. Here, a design methodology combining topology optimization (TO) with honeycomb materials is proposed to achieve lightweight for a typical aircraft double-lug joint structure (DLJS). The initial DLJS is topologically optimized using the variable density method to identify optimal material distribution. The optimized result is then reconstructed into a regular geometric model using the three dimensional (3D) modeling software SolidWorks 2022. In the reconstructed DLJS, the lower stress regions are replaced with honeycomb materials possessing superior mechanical properties or either removed to further enhance stiffness-to-weight ratio. Numerical strength verifications are performed on the final designed DLJS, demonstrating that the maximum stresses designed DLJS remain below the material yield strength under three typical load cases, meeting both strength requirements and safety margins. The mass of the designed DLJS is 38.44 kg, achieving a weight reduction rate of 59.7% compared to the initial DLJS (95.38 kg). Finally, the fabrication feasibility of the designed DLJS is evaluated, and a scaled-down DLJS specimen is fabricated using 3D printing technology with photopolymer resin. This work demonstrates the effectiveness and potential of TO combined with honeycomb materials in lightweighting complex 3D engineering components, providing valuable insights for the lightweight design of intricate 3D structures.

## 1. Introduction

The double-lug joint structure (DLJS) is a key connecting component widely used in aircraft, commonly found in high-load locations such as horizontal tail landing gear hinges, door-to-fuselage attachments, spoiler hinges, wing-fuselage pivot joints, and engine pylons [1,2,3]. Typically paired with bolts or bearings, these structures transmit substantial concentrated loads, and their performance directly affects the safety and reliability of the aircraft. In aerospace engineering, structural lightweight remains one of the core design objectives [4,5]. As a typical load-bearing component, the DLJS must minimize self-weight while ensuring strength and stiffness, thereby enhancing the overall performance and economic efficiency of the aircraft.

Traditional lightweight design methods are often heavily reliant on experience and iterative trial-and-error processes, resulting in lengthy cycles, low efficiency, and often excessive material redundancy due to overly conservative assumptions. With advancements in computer science and technology, structural optimization has emerged as one of the most important methods for developing lightweight and high-performance structures [6,7]. Structural optimization is categorized into size optimization [8], shape optimization [9], and topology optimization (TO) [10,11], based on different types of design variables. Among these, TO serves as an effective design method [12,13]. Within a given design space, load conditions, and constraints, it identifies the optimal material distribution, enabling the conceptualization of high-performance configurations at an early design stage. With the rapid development of additive manufacturing (AM), particularly the maturation of metal 3D printing processes, the traditional limitation of “manufacturing dictating design” is gradually being overcome [14,15]. This breakthrough enables the fabrication of structures with complex geometric configurations. For example, Du et al. [16] employed topology optimization and additive manufacturing methods for optimization design and integrated manufacturing of the cable dome structure, achieving advantages the advantages of a novel shape, a high material utilization rate, and a uniform stress distribution. Pan et al. [17] studied the bracket of aircraft flap as the research object, and the mass of the bracket is reduced by 98% after TO.

Driven by advances in structural optimization theory and AM technology, lightweight material design has become increasingly diverse. Currently prevalent lightweight materials primarily include tetrahedral materials [18], Kagome lattice materials [19], pyramidal lattices [20], honeycomb materials [21,22], triply periodic minimal surfaces (TPMS) [23], and origami-inspired materials [24,25]. Each type exhibits distinct characteristics in terms of specific strength, specific stiffness, and energy absorption. Among them, the hexagonal honeycomb materials with unique geometric configuration exhibits exceptional specific strength, specific stiffness, and shear resistance in the out-of-plane direction [26,27]. This makes it an ideal lightweight structural core material, particularly suited for connecting components subjected to out-of-plane loads. Note that the advantages and disadvantages of these lightweight materials do not provide a detailed analysis in this work, and readers are referred to review papers [28,29] and references therein.

The integration of TO with lightweight material designs has demonstrated significant effectiveness in recent years for the lightweight design of aeronautical structures [30,31]. For example, Liu et al. [32] designed a lightweight sandwich aircraft spoiler using the synthetic of TO, Kagome lattice structures, and high-performance materials, achieving 80% weight reduction compared with the initial design model. Qiu et al. [33] performed a lightweight design of a certain type of aircraft truss structure through topology analysis and size optimization, resulting in a 75.15% reduction in mass compared to the original structure. Using TO, Lin et al. [34] determined the load path for the internal skeleton of the skin-skeleton rudder surface and studied the effect of dot-matrix filling on its flutter and stiffness. Shao et al. [35] found that the topological optimization results, coupled with the functionally graded diamond lattice structures, exhibited the best mechanical performance, yielding a maximum weight reduction of 52.5% for bracket.

The success of these studies demonstrates the significant potential of combining TO with lightweight materials. To explore this potential specifically for a critical connecting component, a lightweight design methodology for a typical aircraft DLJS is presented by leveraging the advantages of TO and honeycomb materials. First, TO is employed to optimize the load path of the initial DLJS. The optimized result is then reconstructed into a regular geometric model using 3D modeling software. Subsequently, the lower stress regions in the reconstructed DLJS are either removed or replaced with honeycomb material to enhance the structure’s stiffness-to-weight ratio. Finally, a numerical model of the final designed DLJS is conducted to validate its strength performance under three typical operating cases, and its manufacturability is discussed.

## 2. Initial Model and Strength Analysis

**Figure 1:**
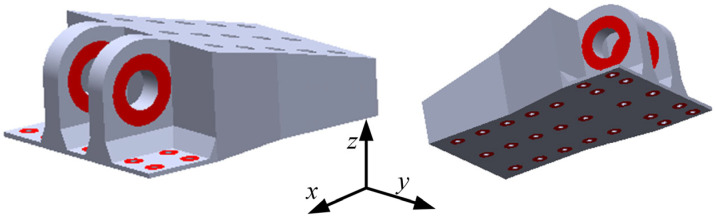
Schematic diagram of the DLJS.

The typical double-lug joint structure (DLJS) of an aircraft serves as a critical connection component. The geometric configuration is illustrated in Figure 1. The overall sizes of the DLJS are approximately 620 mm × 486 mm×245 mm in length, width, and height, with an initial mass and volume of 95.38 kg and 3.45 × 10^−2^ m^3^, respectively. The manufacturing material is a high-strength aluminum alloy, and its material parameters are as follows: density *ρ* = 2760 kg/m^3^, elastic modulus *E* = 70 GPa, Poisson’s ratio *μ* = 0.33, tensile yield strength *σ_y_* = 372 MPa, and compressive yield strength *σ_yc_* = 427 MPa.

The simulations are performed using the commercial finite element (FE) software Abaqus/Standard 6.14 [36]. A mesh convergence study is conducted to ensure the selected element size balanced computational accuracy and efficiency for stress analysis. After convergence analysis, the DLJS is discretized by higher-order tetrahedral elements (C3D10), having a mesh of 216,566 elements with a size of 10 mm.

The practical assembly requirements must be taken into account during the design process. The regions surrounding the double-lug and bolted connections are designated as non-design domains (red areas in Figure 1), but their size is designable, such as the outer radius. The other regions (gray areas in Figure 1) are defined as design domains, allowing for optimization of material distribution. The DLJS is fixed to the base plate by 25 bolts, so the fixed constraints are applied to all bolt holes in the FE models. The load is transmitted to the lugs through the connecting shaft. This study considers three typical load conditions: 1. Both the lugs are subjected to a tensile load of 400 kN in the *x*-direction (*F_x_* = 400 kN); 2. Both the lugs are subjected to a tensile load of 200 kN in the *z*-direction (*F_z_* = 200 kN); 3. Both the lugs are subjected to a compressive load of 200 kN in the *z*-direction (*F_z_* = −200 kN). These load cases were defined based on the industrial design specifications and operational data provided by the Commercial Aircraft Corporation of China, Ltd. (COMAC).

**Figure 2:**
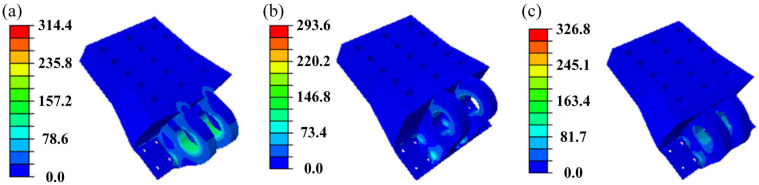
The distribution of von Mises stress for the initial DLJS: (**a**) Case 1, (**b**) Case 2, (**c**) Case 3.

Figure 2 illustrates the distribution of von Mises stress in the initial DLJS under the three typical load cases. The analysis indicates that the maximum stress under all three conditions is concentrated at the edges of the inner bolt holes, located directly below the two lugs. Specifically, the maximum stress values are 314.4 MPa, 293.6 MPa, and 326.8 MPa for Case 1, Case 2, and Case 3, respectively. All these values are below the yield strength (372 MPa) of the aluminum alloy, indicating that the initial DLJS satisfies the strength safety requirement. However, the peak stresses occur only in localized regions, while stress levels in most areas remain significantly below this value. This coexistence of localized stress concentration and overall low stress levels indicates substantial potential for lightweight design.

## 3. Topology Optimization Design

As an advanced structural design method, TO aims to identify the optimal material distribution within a given design space, boundary conditions, and loading conditions to achieve specific performance objectives. For DLJS, the optimization problem falls under the category of continuum TO. Such problems typically use an objective function of maximizing overall structural stiffness (or minimizing compliance), constrained by the permissible material volume fraction within the design domain, to obtain the optimal material distribution. The topology optimization is performed using the commercial software Abaqus in this work.

This study employs the Solid Isotropic Material with Penalization (SIMP) method for TO. In this model, the material density of each element is defined as a design variable ranging from 0 to 1, which allows for intermediate densities. A penalty factor, *p*, is introduced to penalize these intermediate values, thereby driving the optimization solution toward a clear 0–1 (void–solid) distribution. The Young’s modulus of each element in the design domain is parameterized as a function of its design variable to effectively implement this penalization scheme. To penalize intermediate density, the Young’s modulus of the *e*-th element can be expressed as [12]

(1) $$E_{e}=x_{e}^{p}E_{s}$$

where *E_s_* denotes the elastic modulus of the solid element, *x_e_* represents the relative density of each element, and *p* is the penalty factor.

The structure is discretized into *N* finite elements, corresponding to *N* design variables. The global stiffness matrix can be assembled from the elemental stiffness matrices, ***k_e_***, as

(2) $$K=\sum_{e=1}^{N}x_{e}^{p}k_{e}$$

Under given external loads, boundary conditions, and volume constraints, the mathematical model for TO with the objective of minimizing flexibility is [37,38]

(3) $$\begin{array}{l} \mathrm{Min}:\;c(x)=U^{T}KU=\sum_{e=1}^{N}{\left(x_{e}\right)}^{p}u_{e}^{T}k_{e}u_{e}\\ \;\;s.t.:\;\frac{V_{\left(x\right)}}{V_{0}}=f\\ \;\;\;\;\;\;:\;KU=F\\ \;\;\;\;\;\;:\;0<x_{\min}\leq \;x\;\leq 1 \end{array}$$

where *c*(**x**) is the objective function; ***F*** is the global load vector, ***U*** and ***u****_e_* are the global and element displacement vectors, respectively; *V*_(x)_ and *V*_0_ denote the global material volume and the initial design domain volume, respectively; *f* is the material volume fraction; and ***x****_min_* is the vector of minimum relative density.

**Figure 3:**
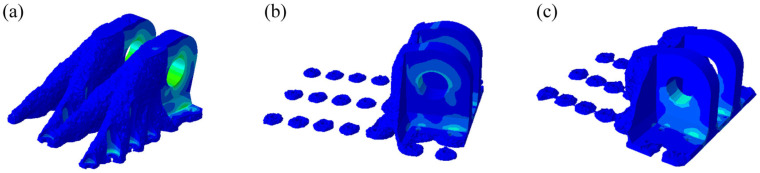
Results of topological optimization for the DLJS: (**a**) Case 1, (**b**) Case 2, (**c**) Case 3.

Based on the aforementioned TO model, optimization calculations are carried out for the DLJS under the three load cases, with a density threshold set at 0.3. The resulting optimal material distribution contours for each case are shown in Figure 3. The results show that material is largely removed from the bottom bolt hole regions farther from the lugs, indicating significant material redundancy. Under all three operating cases, the primary load transfer paths and key bearing regions are concentrated on the lugs and their adjacent regions. In Case 1, the load transfer path exhibits an inclined distribution, extending away from the lugs.

Furthermore, the topology optimization process itself is influenced by the choice of parameters such as the penalty factor and volume constraint. While a sensitivity analysis could reveal the extent of this influence, the primary goal herein was to extract a qualitative and principled load path to inform the conceptual design. The subsequent stages of geometric reconstruction and material selection incorporate engineering judgment and safety factors, which accommodate potential variations in the TO output. Thus, the presented methodology is robust in an engineering sense.

**Figure 4:**
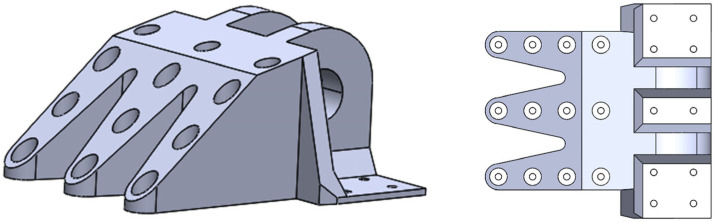
Geometric reconstruction model.

Based on the optimization results (Figure 3), the topology-optimized DLJS is geometrically reconstructed using the 3D modeling software SolidWorks 2022 [39]. As shown in Figure 4, the non-critical load-bearing material around the three bolt holes far from the lugs is removed, achieving a preliminary lightweight design with a mass of 36.23 kg. The material layout around the lugs is preserved, while the bolt hole region is redesigned with regular-shaped features based on the TO results for Case 1 (Figure 3a).

**Figure 5:**
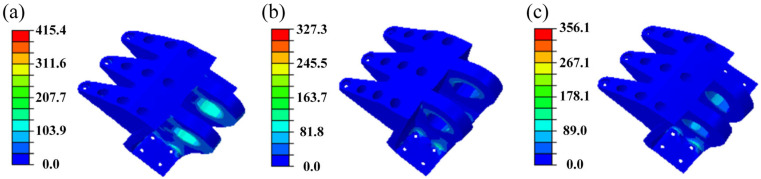
The distribution of von Mises stress for the reconstructed DLJS: (**a**) Case 1, (**b**) Case 2, (**c**) Case 3.

FE analysis is conducted again on the reconstructed DLJS under the three load cases, with the von Mises stress contours shown in Figure 5. The maximum stress remains concentrated in the region directly below the two lugs, but the stress values increase significantly compared to the initial model: 415.4 MPa in Case 1, 327.3 MPa in Case 2, and 356.1 MPa in Case 3. This increase is primarily attributed to the removal of some load-bearing material during the reconstruction process, leading to redistribution of load paths and more pronounced stress concentration effects. Although the maximum stress in Case 1 now exceeds the material’s yield strength, these findings provide a valuable reference for subsequent lightweight design strategies.

## 4. Design of Honeycomb Materials

Honeycomb materials are recognized for their superior strength-to-weight ratio and excellent compressive and shear properties, making them ideal for minimizing weight and material cost in fields like aerospace. These structures consist of an array of thin-walled, typically hexagonal, hollow cells, enabling them to bear significant loads while maintaining minimal weight. Based on the initial lightweight design from TO, a honeycomb material is further incorporated into the aircraft DLJS in this work to achieve a deeper level of weight reduction.

**Figure 6:**
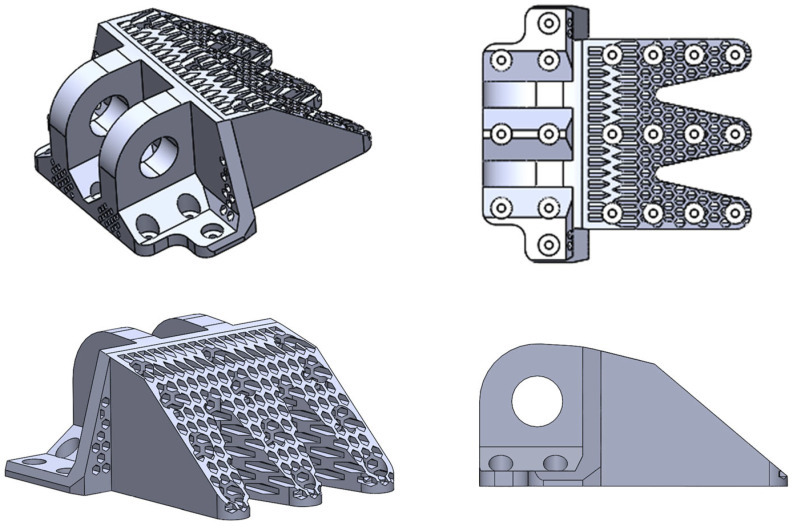
The final designed aircraft DLJS.

To address the stress concentration issue at the bolt hole directly beneath the lugs in the reconstructed DLJS (Figure 5), targeted reinforcement measures are implemented. As illustrated in Figure 6, the 10 mm-thick connecting plate in this critical region is locally thickened to 25 mm. Additionally, supporting material is added to the left and right regions below the lugs to improve the load transfer paths and reduce the peak stress. For other structural regions with relatively low stress levels, particularly those subjected primarily to tensile loading in the *x*-direction, solid material is replaced with hexagonal honeycomb materials. The adopted honeycomb cells have a diameter of 15 mm and a thickness of 6 mm. They are arranged along the primary loading direction (*x*-direction) to fully utilize their anisotropic mechanical properties. This direction is chosen to align with the principal stress direction under the dominant tensile load (Case 1), thereby maximizing the structural stiffness and load-bearing capacity in the most critical loading conditions. Furthermore, this configuration is compatible with common additive manufacturing processes, as the cell walls align with the build direction, reducing the need for excessive support structures and enhancing manufacturability.

The selection of these specific honeycomb parameters is guided by engineering practices and the objective of demonstrating the proposed methodology. It is recognized that these values represent a feasible design choice rather than a unique optimum. Given that the honeycombs are applied in regions of lower stress, a range of cell sizes and thicknesses could potentially be employed without compromising structural integrity. A full-scale optimization of these geometric parameters is not the focus of this study but represents a promising direction for future work to push the weight reduction even further. The primary goal here is to validate the concept that the substitution of solid material with honeycombs in non-critical areas, as guided by topology optimization, is a highly effective strategy for lightweighting.

After material reinforcement in the critical regions of the lugs, the overall structural strength has been enhanced. The regions near the lugs also require redesign. Specifically, the radius of the retained region for the lugs shaft hole is designed to be 95 mm. The two bolt holes at the lower corners of the lugs and the surrounding non-critical material are removed (Figure 6). Additionally, all chamfers and transition regions with potential stress concentrations are treated with rounded corners to effectively prevent new stress concentrations caused by sharp corners.

The aircraft DLJS integrating TO and honeycomb materials is presented in Figure 6. To validate its mechanical performance, a numerical simulation for strength verification is conducted. The FE model became more complex due to the introduction of the honeycomb materials, which is discretized into 4,754,936 tetrahedral elements (C3D10) with a size of 6 mm to ensure computational accuracy. The load and boundary conditions are applied strictly in accordance with the requirements of Cases 1 to 3.

**Figure 7:**
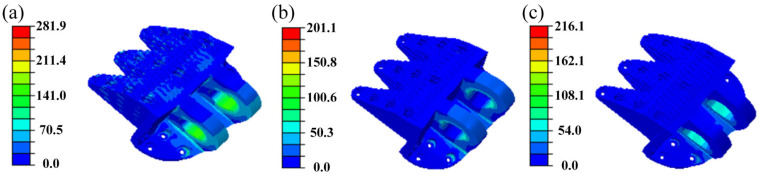
The distribution of von Mises stress for the designed DLJS: (**a**) Case 1, (**b**) Case 2, (**c**) Case 3.

Figure 7 shows the von Mises stress distributions of the designed DLJS under the three load cases. The results indicate that the maximum stress values are 281.9 MPa for Case 1, 201.1 MPa for Case 2, and 216.1 MPa for Case 3. These values are not only significantly lower than the yield strength (372 MPa) of the aluminum alloy, but also represent a notable reduction compared to the maximum stresses in the initial model under the same conditions, as shown in Table 1. This demonstrates that the final designed DLJS fully meets the strength design requirements.

The significant reduction in maximum stress in the final design, compared to the reconstructed model, is primarily a consequence of the local stiffening measures applied to the critical region beneath the lugs (Figure 6). This reinforcement effectively mitigated the stress concentration identified in the previous design stage. The introduction of honeycomb materials in the lower-stress regions primarily served the goal of weight reduction by replacing solid material, with a secondary effect of slightly altering the global load path.

The safety margin is a key indicator for evaluating the safety and robustness of structural design. A reasonably designed safety margin should be positive to ensure a sufficient buffer against uncertainties and unexpected loads. It is defined as the ratio of the difference between the maximum failure load and the design load to the design load. The safety margin for the designed DLJS is

(4) $$n=\frac{\left[\sigma \right]-\sigma^{0}}{\sigma^{0}}=\frac{372-281.9}{281.9}=0.32$$

where [*σ*] represents the yield strength of the material, and *σ*^0^ is the allowable stress.

Based on the computational results, this designed DLJS maintains a positive safety margin under all operating cases. It retains sufficient structural strength reserves even when subjected to extreme loads, fully meeting the anticipated design safety requirements and demonstrating high reliability for engineering applications. Noted that the current study focuses on static strength under typical load cases. For real-world aerospace applications, additional considerations such as fatigue life, dynamic response, and buckling stability under varying operational conditions are critical. These aspects, while beyond the scope of this work, represent important directions for future research.

The mass of the designed aircraft DLJS is reduced to 38.44 kg (Table 1), with the volume correspondingly decreasing to 1.39 × 10^−2^ m^3^. Compared to the initial model (95.38 kg, 3.45 × 10^−2^ m^3^), this corresponds to a remarkable total reduction of 59.70% in both mass and volume. This significant weight-saving effect demonstrates the effectiveness of the integrated design strategy combining TO and honeycomb materials. A reduction of this magnitude is critically important for a typical aircraft connection structure, highlighting the application potential and engineering value of the proposed method in the lightweight design of aerospace structures.

**Figure 8:**
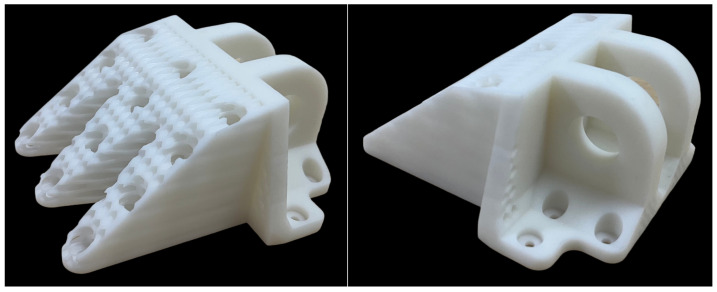
A scaled (4:1) prototype of the designed aircraft DLJS.

Considering the fabrication costs associated with metal AM technology, a scaled-down (4:1) prototype of the designed aircraft DLJS was fabricated using stereolithography (SLA) 3D printing with a photosensitive resin to validate the fabricability, as shown in Figure 8. Noted that the design process fully accounted for the AM process characteristics. Particular attention is paid to avoiding excessively acute angles, minute features, and enclosed cavities, which collectively served to minimize the use of support material and the complexity of subsequent post-processing. The full-scale structure can be fabricated using metal AM processes such as Selective Laser Melting (SLM) or Electron Beam Melting (EBM). Recommended materials include high-strength aluminum alloys or titanium alloys, which offer an excellent strength-to-weight ratios and are widely used in aerospace applications. To ensure manufacturability, the minimum wall thickness should be no less than 3 mm, and the diameter-to-thickness ratio of honeycomb cells should be controlled below 2.5 to avoid printing defects. Post-processing such as heat treatment and hot isostatic pressing is recommended to relieve residual stresses and improve mechanical properties. The support structures should be strategically designed to facilitate removal and minimize surface roughness in critical regions.

It should be noted that the designed lightweight aircraft DLJS is not a globally optimal solution. Firstly, the TO process is prone to converging to local optima, and its results heavily depend on the initial parameters, such as penalty factor, filter radius, and volume constraint, making it difficult to guarantee a globally optimal solution. Second, the modeling process from the TO result to the final integrated honeycomb materials involves a degree of empirical design decisions. These include geometric model reconstruction, reinforcement methods in high-stress regions, and the size and layout of honeycomb cells. Although these engineering-based decisions prove effective, they inevitably introduce subjectivity.

The primary objective of this work is to provide a promising lightweight design methodology for complex structures that integrates TO, honeycomb materials, and AM. This methodology achieves significant weight reduction while maintaining structural integrity. However, for aircraft structures in actual service, it is essential to consider more complex multi-load cases, dynamic responses, fatigue life, and buckling stability constraints. These will all serve as key focuses for future research efforts.

## 5. Conclusions

In this study, a design methodology combining topology optimization (TO) with honeycomb materials is presented to achieve lightweight for a typical aircraft DLJS, leveraging the advantages of AM. The TO first identifies the conceptual layout of the optimal material distribution, which is then converted into a manufacturable CAD model with a regular configuration through geometric reconstruction. The hexagonal honeycomb materials are introduced to replace solid material in regions with relatively low stress levels.

Results demonstrate that the designed aircraft DLJS weighs as low as 38.44 kg, achieving a weight reduction of 56.94 kg, with a significant weight reduction of 59.7% compared to the initial DLJS (95.38 kg). The lightweight designed DLJS not only meets static strength requirements under three typical load cases but also exhibits lower maximum stress levels than the initial model, i.e., the structural strength satisfies requirements while maintaining a safety margin. The fabrication feasibility of the designed DLJS is evaluated, and a scaled-down DLJS is fabricated using 3D printing technology with photopolymer resin. Looking towards industrial adoption, the design is compatible with high-strength metal additive manufacturing processes such as Laser Powder Bed Fusion (LPBF). Although the current manufacturing cost for such processes is relatively high, it is economically viable for high-value, critical aerospace components like the DLJS. The substantial weight reduction achieved directly translates into fuel savings and performance gains over the aircraft’s lifecycle.

The lightweight design of irregular 3D structures, commonly encountered in engineering practice, entails complexities that extend far beyond those of their 2D structures. These challenges are not only in the enormous computational demands but also in the post-processing of optimization results and their translation into detailed, manufacturable designs. The successful lightweight design of the typical aircraft double-lug joint structure provides valuable experience and reference for extending established optimization techniques to more complex irregular 3D structures.

## Figures and Tables

**Table 1:** Comparison of mass and maximum von Mises stress for different design stages.

Design Stage	Mass (kg)	Max Stress Case 1 (MPa)	Max Stress Case 2 (MPa)	Max Stress Case 3 (MPa)
Initial DLJS	95.38	314.4	293.6	326.8
Reconstructed DLJS	36.23	415.4	327.3	356.1
Designed DLJS	38.44	281.9	201.1	216.1

## Data Availability

The original contributions presented in this study are included in the article. Further inquiries can be directed to the corresponding authors.
